# Expression of microRNA-223 and microRNA-214 in gingival crevicular fluid of smoker and nonsmoker periodontitis patients, an observational diagnostic accuracy study

**DOI:** 10.1007/s00784-024-05844-7

**Published:** 2024-08-10

**Authors:** Maha AbdelKawy, Nayroz Abdel Fattah Tarrad, Olfat Gamil Shaker, Sandy Hassan

**Affiliations:** 1 Oral Medicine and Periodontology Department, Faculty of Dentistry, Beni-Suef University, Beni-Suef, Egypt; 2 Oral Medicine and Periodontology Department, Faculty of Dentistry, Fayoum University, Fayoum, Egypt; 3 Medical Biochemistry and Molecular Biology Department, Faculty of Medicine, Cairo University, Cairo, Egypt; 4 Oral Medicine and Periodontology Department, Faculty of Dentistry, Fayoum University, Cairo, Egypt

**Keywords:** Periodontitis, MicroRNA, Smoker, GCF, Biofluids

## Abstract

**Objective:**

Periodontitis is a multifactorial disease that affects a wide range of populations. However, its pathogenesis remains unclear. miRNAs are now considered potential diagnostic markers for many inflammatory diseases. Thus, the aim of this study was to assess the expression of microRNA-223(miRNA-223) and microRNA-214 (miRNA-214) in gingival crevicular fluid (GCF) of smoker and nonsmoker with periodontitis.

**Materials and methods:**

We conducted a prospective study among 42 participants: 14 healthy controls, 14 nonsmoker periodontitis participants, and 14 smokers with periodontitis. Eligibility criteria for inclusion were consecutive adults, aged 20–60 years, with stage III periodontitis grade B/C and no systemic diseases. All consenting participants had gingival crevicular fluid samples collected after diagnosis to assess miRNA-214 and -223 by quantitative real-time polymerase chain reaction assay.

**Results:**

ROC curve analyses for the non-smoker periodontitis group showed that miR-214 as a predictor in comparison to miR-223 had higher sensitivity [92.86%-64.29%], same specificity [100%], and a significantly higher area under the curve [0.974–0.796] respectively (*p* = 0.036). As for the smoker periodontitis group, a ROC curve with miR-214 as predictor in comparison to miR-223 had higher sensitivity [100%-71.43%], same specificity [100%], and a non-significantly higher area under the curve [1–0.872], respectively (*p* = 0.059).

**Conclusion:**

Both miRNA-214 and 223 are reliable potential diagnostic markers for periodontitis, with miRNA-214 being more accurate for smokers with periodontitis.

**Clinical relevance:**

Both miRNA-214 and 223 could be considered for potential chair-side diagnostics, by simply collecting GCF detecting the disease in its first steps and aid in preventing unrepairable damage.

## Introduction

Periodontal diseases and conditions are a span of diseases that affect the tooth supporting tissues. It is a widely occurring chronic inflammatory disease induced by oral biofilm, primarily associated with plaque biofilm dysbiosis, leading to the destruction of alveolar bone. Periodontitis is a multifactorial disease affecting a wide range of populations and it is responsible for a significant proportion of edentulism and masticatory dysfunction [1, 2]. It is a distinctive disease in which the defensive process acts as the cause of most tissue destruction, where there is a complex interplay between bacteria, inflammatory host response, and environmental factors [3, 4].

Smoking plays a significant role in periodontitis pathogenesis. It significantly affects the prognosis of the disease; therefore, it has been added as a modulating factor in the grading system of the latest periodontal disease classification [4]. Tobacco and its derivatives play a role in increasing the pathogenicity of periodontal pathogens, making the host highly susceptible to infection. Moreover, it hastens disease progression by increasing periodontal destruction and delaying treatment [1]. Although smoking increases the risk of periodontal disease, simultaneously, it reduces the clinical signs such as gingival bleeding and inflammation. This leads to hiding the early signs and symptoms of the disease. [5]. In addition, increased number of pocket (sites and depths) and increased frequency of bone loss were found to be greater in smokers then in non-smokers [6]. Smoking doesn't affect only periodontal tissue through direct effect of nicotine as a vasoconstrictor, but also, on the long term, it impairs the periodontal tissue vasculature [7].

MiRNAs are short non-coding RNAs composed of 21–23 nucleotides. They negatively affect gene expression by interacting with messenger RNA and disrupting their translation. They have diverse effects on different biological cascades, including inflammation. Their expression levels differ according to tissue health or disease [8, 9]. Earlier, it was assumed that they would act only within their synthesizing cells, which was imprecise. miRNAs leave their synthesizing cells through extracellular vesicles and exosomes, defusing into the blood and reaching other cells. They can act on their recipient cells and cause alterations in gene expression [10]. Abnormal expression of miRNAs has been found to be related to many diseases. miRNA-223 was found to play a role in inflammatory and autoimmune diseases. It showed over expression in CD4 + naive T-lymphocytes of rheumatoid arthritis patients [11]. Moreover, miRNA-214 was found to be down regulated in cervical cancer cells [12]. MiR-214 suppressed oxidative stress in diabetic nephropathy [13].

Recently, miRNAs have been used as biomarkers for the diagnosis of various diseases and for prognostic purposes. The expression of different miRNA profiles has been tested in various studies [14–16]. Amaral et al. compared chronic and aggressive periodontitis by studying different miRNA expression profiles in gingival tissues. They found no differences between the two periodontitis groups [17]. In addition, Lee et al. investigated 84 miRNA expression in the saliva of participants with aggressive periodontitis and compared them with those of healthy participants They identified four different salivary miRNAs (hsa-let-7a-5p, hsa-let-7f-5p, hsa-miR-181b-5p, and hsa-miR-23b-3p) being down regulated in aggressive periodontitis and considered this as first step of noninvasive screening and diagnostic assay for aggressive periodontitis [18]. Moreover, a recent study assessed the expression of six miRNAs in the saliva of smoker and nonsmoker with periodontitis. Their results suggested the increase of the salivary miR-146a, miR-146b, miR142-3p,miR-155, and miR-203 gene expressions with the progression of periodontal disease, and was unaffected by periodontal treatment. They added that smoking may upregulate salivary miR-142-3p levels in the periodontal health and disease. [19]. Smoking, particularly the nicotine, was found to highly affect miRNAs expression (up to two folds) where it decreased regeneration of periodontal stem cells delaying healing in smoker periodontitis patients [15].

Gingival crevicular fluid (GCF) is considered a “window” of the periodontium, and its production is directly related to signs of gingival inflammation. As a fluid in close proximity to periodontal tissue, GCF has been the principal target for exploring biomarkers for periodontal diseases [20]. MicroRNA- 223 was identified in healthy and inflamed gingival tissues and was found to be significantly upregulated in inflamed gingival tissues [21]. Another study also showed relative increase in quantification levels of of microRNA- 223 in serum and GCF in chronic periodontitis diabetic patients [22]. Whereas microRNA-214 was investigated in gingival tissues of diabetic periodontitis patients. Their levels were found to be upregulated [23].

To the best of our knowledge miRNA-214 was not measured in the GCF of periodontitis patients before. Both molecules were not examined in smoker periodontitis patients. There is a scarcity of research in the literature investigating miRNA expression in GCF in periodontitis and its relation to smoking. Hence, the aim of the current study was to assess the expression of microRNA- 223 and microRNA-214 in GCF of smoker and nonsmoker participants with periodontitis.

## Methodology

### Subjects’ recruitment

This was a prospective, observational, case–control study. This study was approved by the research ethics committee (ID number*# REC-FDBSU/05012023–04/AM* and was performed in full accordance with the World Medical Association Declaration of Helsinki 1975, revised in 2013. The study was retrospectively registered on clinical trial.gov with registration number (NCT06064799).

### Eligibility criteria include

#### Inclusion criteria

- Both sexes with age range from 20 to 60 years.
- Participants agreeing to consent.
- Systemically healthy participants
- Generalized periodontitis stage III with grade B/C (for periodontitis group).
- Current Smoker with Generalized periodontitis stage III with grade C (for smoker’s periodontitis group).

#### Exclusion criteria

- Periodontitis patients having periodontal treatment recently (past 6 months).
- Pregnancy, lactation, contraceptive pills.
- Anti‑inflammatory/immunosuppressive drugs.
- Patients having any appliances that would locally affect the periodontal condition.

**Fig. 1:**
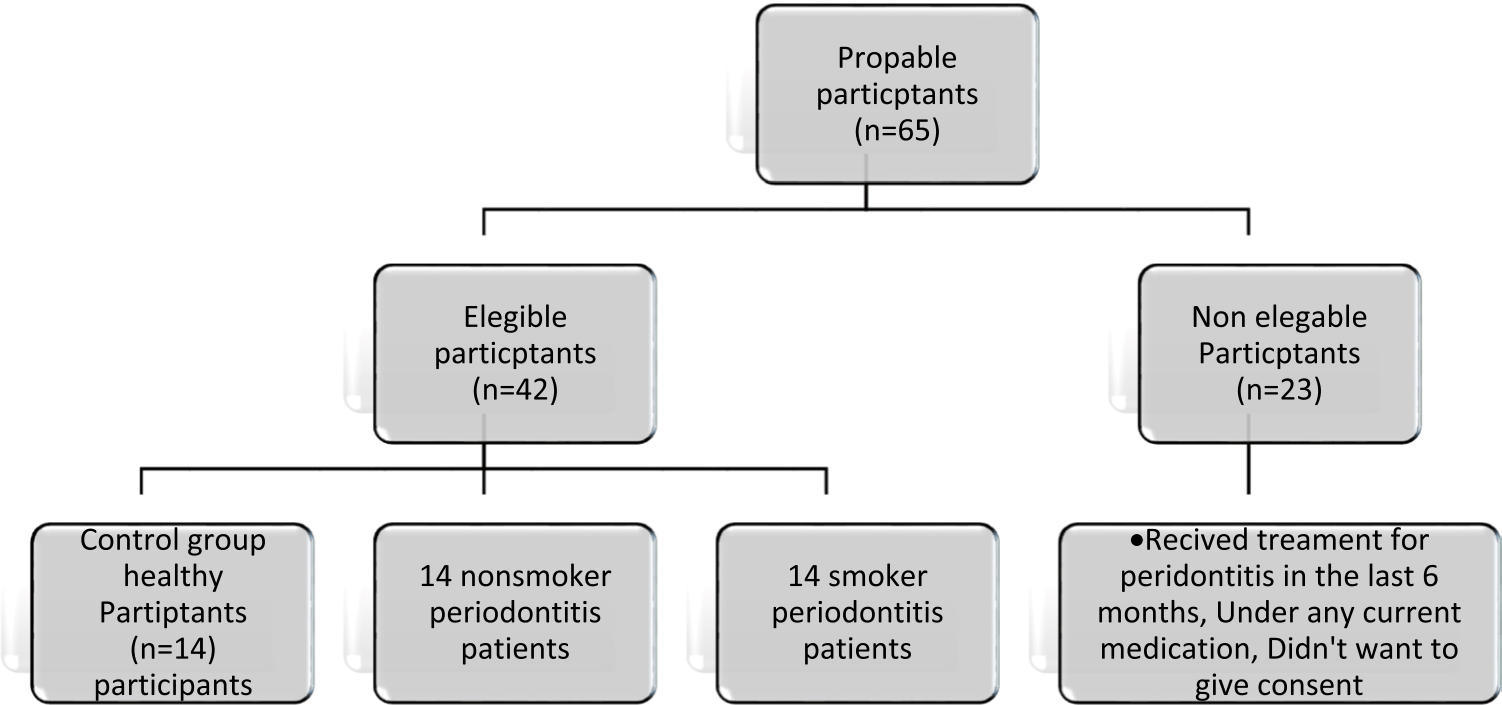
Flow chart for participants

A total of forty-two systemically healthy participants were recruited consecutively from the outpatient clinic of the department of periodontology with the starting date of January 2023 until June 2023. The three groups were categorized as follows: 14 healthy nonsmokers volunteers as control group, 14 periodontitis participants with stage III grade B/C Periodontitis who never smoked as nonsmoker periodontitis group, and 14 smoker periodontitis participants with stage III grade C Periodontitis as smoker periodontitis group (Fig. 1). Periodontitis was diagnosed according to the new classification of periodontal disease. The smokers in the periodontitis group were assigned as grade C as they smoked more than ten cigarettes per day [1]. Clinical periodontal parameters were registered by single examiner using William’s graduated periodontal probe; plaque index (PI) [24], probing depth (PD) [25], bleeding on probing (BOP) [26] and clinical attachment loss (CAL) [27], to reach proper diagnosis. All these clinical parameters were assessed for each tooth at 6 sites (mesio-buccal/lingual, disto-buccal/lingual, mid-buccal/lingual), and recorded for all included participants. PI was measured according to presence/ absence of the supragingival biofilm by sweeping motion of the periodontal probe around surfaces of all teeth [18]. Bleeding on probing was measured as present, i.e. recorded when bleeding occurs. Bone loss was radiographically assessed. After diagnosis, participants received phase one periodontal therapy (scaling and root planning), were given oral hygiene instructions, and were assigned for follow-up. Written informed consent was obtained from all participants and healthy volunteers.

Diagnosis of periodontitis participants followed the new classification of periodontal disease [2] where CAL is the main factor deciding the stage of periodontitis (≥ 6 mm in stage III) and the grade decided by indirect evidence of the disease progression through percentage of bone loss assessed radiographically to age and the case phenotype (presentation of plaque). In the smoker periodontitis group, the quantity of smoked cigarettes is considered a grade modifier changing the grade into a grade C as all smokers smoked more than 10 cigarettes per day. The control group included healthy participants who had clinically healthy gingiva (PD ≤ 3 mm, nearly zero PI, zero CAL, and less than 10% BOP) [2].

### GCF sample collection

The GCF samples were collected in a morning session by trained periodontist using absorbent paper strips. Before inserting the paper strips using a cotton pallet, any supragingival plaque was removed and saliva was dried. In the same visit, samples were obtained from single rooted teeth by using strips from pockets with the deepest readings to collect undiluted GCF. Upon collection and after giving each sample a serial number, the samples were frozen at -80^O^ C in the biochemistry laboratory for analysis [21].

### MiRNA analysis

GCF samples were analyzed separately. Total RNA was isolated from GCFs using a mirVana miRNA isolation kit (Applied Biosystems). We used a spectrophotometer to measure RNA concentrations. Copy DNA (cDNA) was generated using the Prime Script RT reagent kit (Applied Biosystems) in a 20 μl final reaction volume containing 0.5 μg RNA, 0.5 μl Prime-Script RT enzyme mix, 4 μl 5 × PrimeScript buffer, and 1 μl RT primer. The mixture was incubated at 42 °C for 60 min, and then at 85 °C for 5 min.

Quantitative real-time polymerase chain reaction (PCR) assays were performed to assess miRNA-233 and miRNA-214 expression using SYBR Premix Ex Taq (Applied Biosystems). Primers for miRNA-233 and miRNA-214 and the endogenous control U6 snRNA were obtained from Applied Biosystems. We then measured each miRNA using the Step One Plus System supplied by Applied BioSystem (USA). Denaturation cycling was performed at 95 °C for 10 min, followed by 45 cycles of denaturation (95 °C for 15 s), annealing (60 °C for 30 s) and extension (72 °C for 1 min). The relative expression levels of miRNA-233 and miRNA-214 were calculated and normalized using the 2-ΔΔCt method relative to the U6 small nuclear RNA [28].

### Sample size calculation

Sample size calculations were performed by designing a power analysis to have adequate power to apply a statistical test of the null hypothesis that there was no diagnostic ability of the tested markers. By adopting an alpha level (α) of (0.05), a (β) level of (0.2) (i.e., power = 80%), a null hypothesis value of (0.6), and receiver operating characteristic (ROC) value of (0.87) acquired from the results of a previous study^1^ [22], the minimal required sample size (n) was found to be (42) cases (i.e., 14 cases per group). The sample size calculation was performed using MedCalc® Statistical Software version 20.019.^2^

### Statistical analysis

Categorical data are presented as frequency and percentage values and were analyzed using Fisher’s exact test. Numerical data are presented as mean and standard deviation (SD) values. They were tested for normality using the Shapiro–Wilk test. Non-parametric data (miR-214-fold change (Fc) and miR-223 (Fc)) were analyzed using the Kruskal–Wallis test followed by Dunn’s post hoc test with Bonferroni correction. Other numerical data were normally distributed and analyzed using one-way ANOVA variance followed by Tukey’s post hoc test. ROC curve analysis was performed to determine diagnostic accuracy. The ROC curves were compared using the z-test. The significance level was set at *p* < 0.05, for all tests. Statistical analysis was performed using the R statistical analysis software, version 4.1.3, for Windows.^3^

## Results

The study included 42 cases (14 cases per group). There were seven (50.0%) males and seven (50.0%) females in the non-smoker periodontitis and control groups, respectively. In the smoker-periodontitis group, there were 9 (64.3%) males and 5 (35.7%) females. The mean age of the cases in non-smoker periodontitis group was (44.00 ± 8.40) years, in smoker-periodontitis group it was (47.43 ± 9.57) years while in the control group it was (42.00 ± 2.19) years. There were no significant differences between the tested groups in terms of sex (*p* = 0.792) and age (*p* = 0.164), Table 1.

**Table 1:** Intergroup comparison of demographic data

Parameter			Nonsmoker-periodontitis (*n* = 14)	Smoker-periodontitis (*n* = 14)	Control (*n* = 14)	Statistic	*p*-value
Gender	Male	*n*	7	9	7	**0.77**	**0.792**
		*%*	50.0%	64.3%	50.0%		
	Female	*n*	7	5	7		
		*%*	50.0%	35.7%	50.0%		
Age (years)	Mean ± SD		44.00 ± 8.40	47.43 ± 9.57	42.00 ± 2.19	**1.90**	**0.164**

*SD *standard deviation

Results of intergroup comparisons presented in Table 2 showed that for all tested clinical parameters, both tested groups had significantly higher values than the control group, with the difference between them being non-statistically significant.

**Table 2:** Intergroup comparison of clinical parameters

Parameter	Mean ± SD			t-value	*p*-value
	Non-smoker periodontitis (*n* = 14)	Smoker-periodontitis (*n* = 14)	Control		
PD (mm)	5.88 ± 0.45^A^	5.91 ± 0.58^A^	1.50 ± 0.55^B^	**14.53**	< 0.001*
CAL (mm)	6.65 ± 0.66^A^	6.54 ± 0.74^A^	0.00 ± 0.00^B^	**14.71**	< 0.001*
BOP %	90.14 ± 4.50^A^	91.04 ± 11.34^A^	3.83 ± 1.72^B^	**15.33**	< 0.001*
Plaque score (%)	86.33 ± 5.43^A^	89.19 ± 5.04^A^	0.67 ± 0.52^B^	**15.99**	< 0.001*

Different superscript letters indicate a statistically significant difference within the same horizontal row

*PD* probing depth, *CAL* clinical attachment loss, *BOP* bleeding on probing

*significant (*p* < 0.05)

**Fig. 2:**
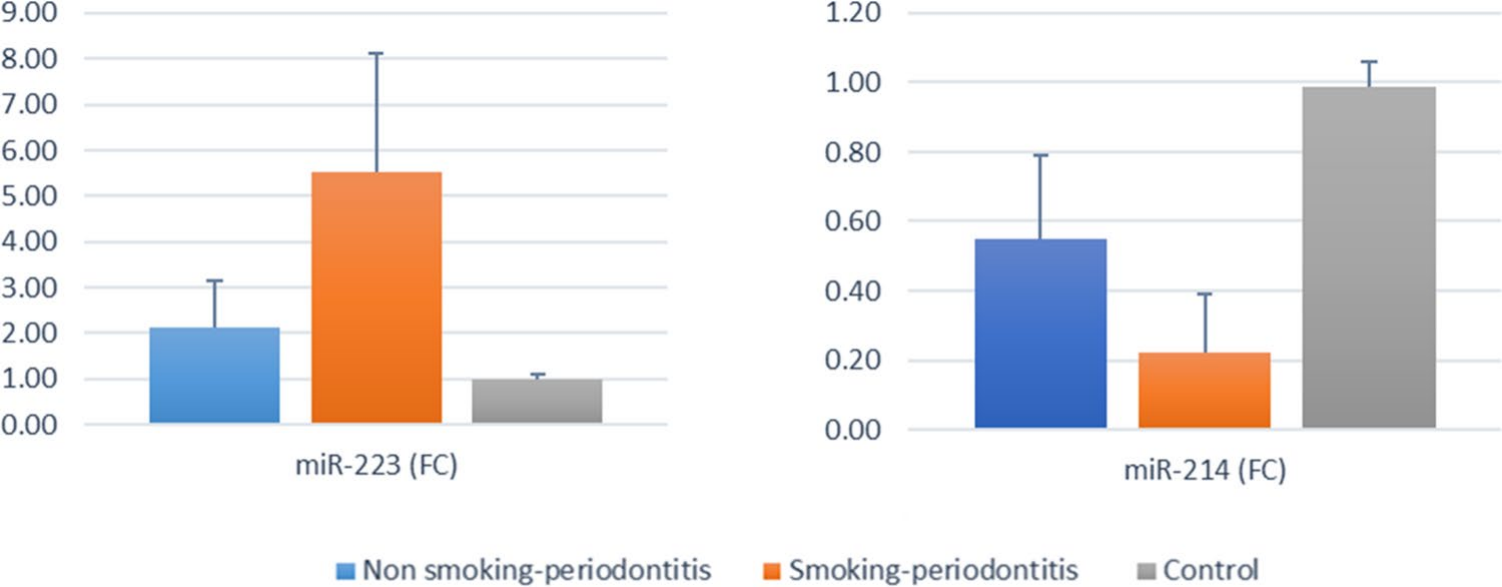
Bar chart showing mean and standard deviation (error bars) for miR-214 (FC) and for miR-223 (FC)

The results of intergroup comparisons presented in Table 3 showed that for both markers, there was a significant difference between the tested groups (*p* < 0.001). For miR-214 (FC), post-hoc pairwise comparisons showed that the control group had a significantly higher value than the other groups (*p* < 0.001). miR-223 (FC) showed a significantly lower value in the control group than in the smoker-periodontitis group (*P* < 0.001). The mean and standard deviation values for the miR-214 (FC) and miR-223 (FC) levels are presented in Fig. 2.

**Table 3:** Intergroup comparison of biochemical parameters

Parameter	Mean ± SD			h-value	*p*-value
	Nonsmoker-periodontitis (*n* = 14)	Smoker Periodontitis (*n* = 14)	Control (*n* = 14)		
miR-214 (FC)	0.55 ± 0.24^B^	0.22 ± 0.17^B^	0.99 ± 0.07^A^	**31.32**	< **0.00**1*
miR-223 (FC)	2.12 ± 1.03^AB^	5.51 ± 2.62^A^	0.98 ± 0.13^B^	15.09	< **0.00**1*

Different superscript letters indicate statistically significant differences within the same horizontal row

*significant (*p* < 0.05)

Results for the correlations between different clinical parameters and miR-214 (FC) level presented in Table 4 showed that for the non-smoker periodontitis group, there was a negative strong correlation with mean pocket depth which was statistically significant (rs = -0.706, *p* = 0.005). Overall, there was a negative weak correlation with the same parameter that was also statistically significant (rs = -0.384, *p* = 0.043). Other correlations were not statistically significant (*p* > 0.05).

**Table 4:** Clinical  parameters correlations with miR-214 (FC)

Group	Parameters Mean ± SD	Correlation coefficient [95% CI]	z-value	*p*-value
Nonsmoker-periodontitis (*n* = 14)	*PD (mm)*	**-0.706 (-0.900:-0.281)**	**776.25**	0.005*
	*CAL (mm)*	**-0.492 (-0.811:0.052)**	**678.98**	**0.074**
	*BOP %*	**-0.355 (-0.745:0.217)**	**616.42**	**0.213**
	*Plaque score (%)*	**-0.349 (-0.742:0.223)**	**613.87**	**0.221**
Smoker—periodontitis (*n* = 14)	*PD (mm)*	**-0.247 (-0.687:0.327)**	**567.24**	**0.395**
	*CAL (mm)*	**-0.282 (-0.707:0.292)**	**583.41**	**0.328**
	*BOP %*	**0.494 (-0.049:0.812)**	**230.19**	**0.072**
	*Plaque score (%)*	**0.493 (-0.051:0.811)**	**230.79**	**0.073**
*Overall (n* = 28*)*	*PD (mm)*	**-0.384 (-0.662:-0.013)**	**5058.58**	0.043*
	*CAL (mm)*	**-0.208 (-0.540:0.179)**	**4415.46**	**0.287**
	*BOP %*	**0.069 (-0.313:0.431)**	**3403.52**	**0.729**
	*Plaque score (%)*	**-0.095 (-0.452:0.289)**	**3999.80**	**0.632**

*PD* probing depth, *CAL* clinical attachment loss, *BOP* bleeding on probing

^*^significant (*p* < 0.05)

Results for the correlations between different clinical parameters and miR-223 (FC) level presented in Table 5 showed that for the non-smoker periodontitis group, there was a negative moderate correlation with bleeding on probing which was statistically significant (rs = -0.565, *p* = 0.035). Overall, there was also a negative moderate correlation with the same parameter that was statistically significant (rs = -0.416, *p* = 0.028). Other correlations were not statistically significant (*p* > 0.05).

**Table 5:** Clinical parameters Correlations with miR-223 (FC)

Group	Parameter Mean ± SD	Correlation coefficient [95% CI]	z-value	*p*-value
Nonsmoker-periodontitis (*n* = 14)	*PD (mm)*	**-0.354 (-0.745:0.218)**	**615.89**	**0.215**
	*CAL (mm)*	**-0.270 (-0.700:0.304)**	**577.90**	**0.350**
	*BOP %*	**-0.565 (-0.843:-0.049)**	**712.09**	0.035*
	*Plaque score (%)*	**-0.474 (-0.803:0.076)**	**670.56**	**0.087**
Smoker—periodontitis (*n* = 14)	*PD (mm)*	**-0.053 (-0.568:0.491)**	**479.29**	**0.856**
	*CAL (mm)*	**0.354 (-0.218:0.745)**	**294.05**	**0.215**
	*BOP %*	**-0.435 (-0.785:0.124)**	**653.14**	**0.120**
	*Plaque score (%)*	**-0.175 (-0.646:0.392)**	**534.58**	**0.550**
Overall (*n* = 28)	*PD (mm)*	**-0.072 (-0.434:0.309)**	**3917.44**	**0.715**
	*CAL (mm)*	**0.069 (-0.312:0.431)**	**3400.13**	**0.725**
	*BOP %*	**-0.416 (-0.683:-0.051)**	**5174.31**	0.028*
	*Plaque score (%)*	**-0.255 (-0.573:0.131)**	**4584.24**	**0.191**

*PD* probing depth, *CAL* clinical attachment loss, *BOP* bleeding on probing

^*^significant (*p* < 0.05)

**Fig. 3:**
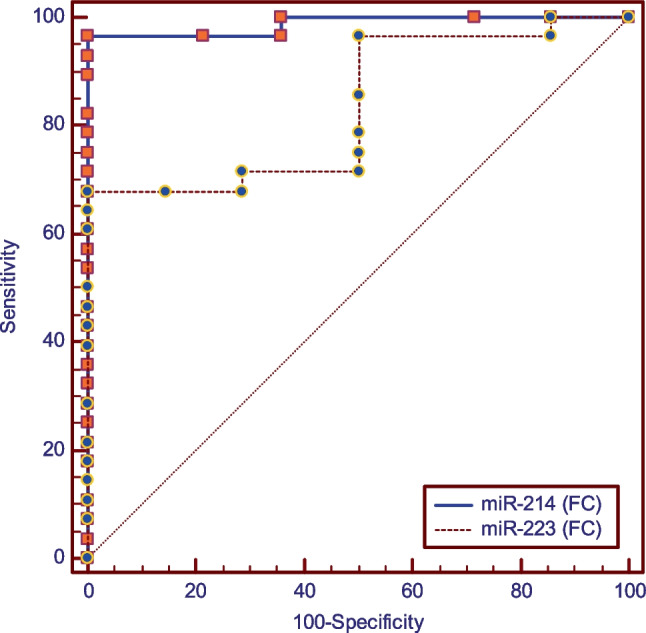
ROC curve analysis for both periodontitis groups together

Results of ROC curve analyses presented in Fig. 3 and Table 6 constructed with both periodontitis groups as positives showed that a ROC curve with miR-214 (FC) as a predictor in comparison to miR-223 (FC), had higher sensitivity [96.43%-67.86%], same specificity [100%], and significantly higher area under the curve [0.987–0.834] (*p* = 0.008).

**Table 6:** ROC curve analysis for both periodontitis groups together

Parameter		miR-214 (FC)	miR-223 (FC)	AUC difference [95%CI]	SE	z-value	*p*-value
Cut off point		≤ **0.83**	≥ **1.33**	**0.153[0.041:0.265]**	**0.06**	**2.68**	0.008*
Sensitivity [95%CI]		**96.43 [81.70–99.90]**	**67.86 [47.6—84.1]**				
Specificity [95%CI]		**100.0 [76.80–100.0]**	**100.0 [76.8—100.0]**				
Accuracy [95%CI]		**97.62% [92.86%-100.00%]**	**80.95% [69.05%:90.48%)]**				
AUC	AUC [95%CI]	**0.987 [0.893 -1]**	**0.834[0.687–0.931]**				
	SE	**0.01**	**0.06**				

*AUC* area under the curve, *FC* fold change, *CI* confidence interval, *SE* standard of error

^*^Significant (*p* < 0.05)

**Fig. 4:**
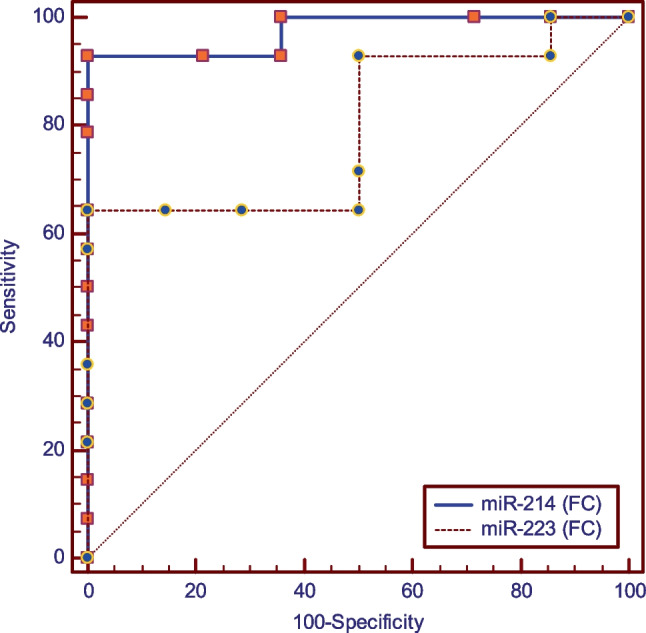
ROC curve for nonsmoker periodontitis group

Results of ROC curve analyses presented in Fig. 4 and Table 7 constructed with non-smoker periodontitis group as positives showed that an ROC curve with miR-214 (FC) as a predictor in comparison to miR-223 (FC), had higher sensitivity [92.86%-64.29%], same specificity [100%], and significantly higher area under the curve [0.974–0.796] (*p* = 0.036), respectively.

**Table 7:** ROC curve analysis for non-smokers periodontitis and healthy control groups

Parameter		miR-214 (FC)	miR-223 (FC)	AUC difference [95%CI]	SE	z-value	*p*-value
Cut off point		≤ **0.85**	≥ **1.31**	**0.179 [0.011:0.346]**	**0.09**	**2.09**	0.036*
Sensitivity [95%CI]		**92.86 [66.10—99.80]**	**64.29 [35.10—87.20]**				
Specificity [95%CI]		**100.0 [76.80—100.0]**	**100.0 [76.80—100.0]**				
Accuracy [95%CI]		**96.43% [89.29%:100.00%]**	**82.14% [71.43%:92.86%]**				
AUC	AUC [95%CI]	**0.974 [0.832 -1]**	**0.796[0.687–0.931]**				
	SE	**0.03**	**0.09**				

*AUC* area under the curve, *FC* fold change, *CI* confidence interval, *SE* standard of error

^*^Significant (*p* < 0.05)

**Fig. 5:**
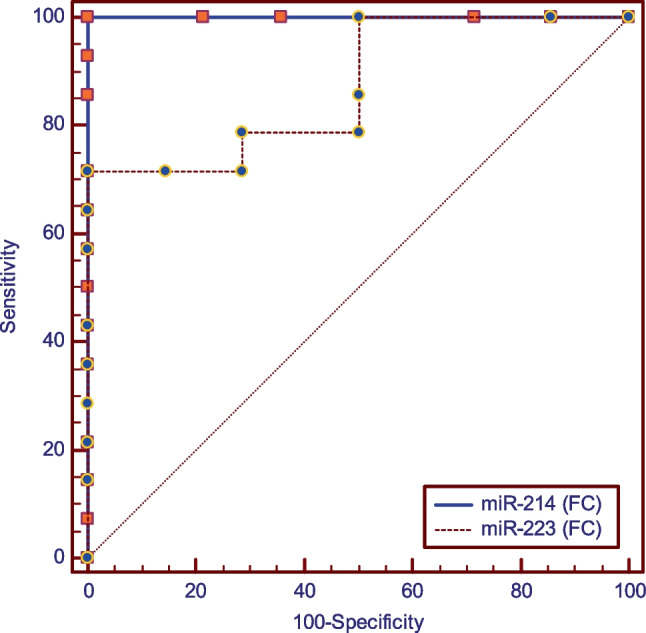
ROC curve for smoker-periodontitis group

Results of ROC curve analyses presented in Fig. 5 and Table 8 constructed with the smoker periodontitis group as positives showed that an ROC curve with miR-214 (FC) as a predictor in comparison to miR-223 (FC), had higher sensitivity [100%-71.43%], same specificity [100%], and a non-significantly higher area under the curve [1–0.872] (*p* = 0.059).

**Table 8:** ROC curve analysis for smokers periodontitis and healthy control groups

Parameter		miR-214 (FC)	miR-223 (FC)	AUC difference [95%CI]	SE	z-value	*p*-value
Cut off point		≤ **0.78**	≥ **1.36**	**0.128 [-0.004:0.260]**	**0.07**	**1.89**	**0.059**
Sensitivity [95%CI]		**100.0 [76.80—100.0]**	**71.43 [41.90- 91.60]**				
Specificity [95%CI]		**100.0 [76.80—100.0]**	**100.0 [76.80—100.0]**				
Accuracy [95%CI]		**100.00% [100.00%:100.00%]**	**85.71% [75.00%:96.43%]**				
AUC	AUC [95%CI]	**1 [0.877 -1]**	**0.872 [0.692–0.968]**				
	SE	**0.00**	**0.07**				

*AUC* area under the curve, *FC* fold change, *CI* confidence interval, *SE* standard of error

^*^Significant (*p* < 0.05)

## Discussion

To date, the pathogenesis of periodontal disease has not been fully understood. Three main factors interact with each other: pathogenic bacteria, host response, and environmental factors. Smoking is one of the major modifying risk factors affecting the grading of periodontitis and consequently, its prognosis [29]. The search for biomarkers in GCF is now the focus of many studies. It is said that evaluating GCF or gingival tissue samples would be more reliable than serum for testing miRNA gene expression involvement and association with periodontal disease [13]. Hereupon we investigated the GCF of nonsmoker periodontitis patients, smoker periodontitis patients, and healthy participants to assess miRNA-223 and miRNA-214 expression, in an effort to confirm their potential as biomarkers for diagnosing periodontal disease.

We found no statistical difference among the three groups in terms of demographic data. As for the clinical parameters, there were no statistical differences between either periodontitis groups, while both showed higher values than the control group*.* This reflects the effect of periodontitis on the clinical level giving all signs of inflammation. No difference found between both periodontitis groups (smoker and nonsmoker) which may be due to their selection in the same stage of periodontitis.

Our results for miRNA-223 gene expression were found to be overexpressed in the smoker periodontitis group, followed by the non-smoker periodontitis group, with the lowest expression in the control group. In addition, the diagnostic accuracy was 82.14% for the non-smoker periodontitis group and 85.71% for the smoker periodontitis group. Thus, miRNA-223 expression can indicate the existence of periodontal disease, especially in smokers with periodontitis, and reflects its role in periodontal disease pathogenesis. This could be related to the miRNA-223 effect on osteogenesis by depriving bone formation and increasing inflammation through cell apoptosis [30]. As for its highest levels in smoker periodontitis group, it could be explained by the effect of nicotine. Nicotine was found to influence 225 different miRNAs by altering their gene expression up to twofold; some of which are related to osteogenesis [31]. Cigarette Smoking was found to negatively affect the regenerative potential of periodontal ligament stem cells, leading to delayed healing in smoker periodontitis [31, 32].

Concurrently, recent research explored the expression of miRNA in the serum and GCF of participants with chronic periodontitis, chronic periodontitis coexisting with diabetes, and those in good periodontal health. The study revealed that miRNA-223 and miRNA-200b were notably overexpressed in the serum and GCF of subjects with periodontitis and periodontitis with diabetes. This aligns with our findings regarding miRNA-223 where we found its upregulation in both periodontitis groups. The study concluded that miRNA-223, miRNA-200b, and miRNA-203 are implicated in the pathogenesis of the disease, correlating with clinical parameters and inflammatory mediators (TNF alpha and Interleukin-10). This supports our earlier proposition about the role of miRNA-223 in the pathogenesis of the disease where it increases the inflammatory process [22]. In line with our findings, miRNA-223 was found significantly upregulated during inflammation and decreased during osteogenesis in periodontal cells [32]. Thus, the overexpression of miRNA-223 negatively affected periodontally derived osteogenesis. Additionally, it was reported that the receptor genes FGFR2 and TGFBR2 are targeted by miRNA-223. Their suppression hinders osteogenic differentiation of periodontal cells [32]. We found negative moderate correlation between miR-223 and bleeding on probing in both periodontitis groups pooled together, it was also significantly found in the nonsmoker periodontitis group. This could be attributed to the small sample size and the sensitivity of correlation of clinical parameters tests to sample size.

As for miRNA-214, our results showed that miRNA-214 was highly expressed in the control group. Its expression was lower in the non-smoker periodontitis group and lowest in the periodontitis smoker group. Its diagnostic accuracy was 100% for smoker periodontitis and 96.43% for nonsmokers. This implies that miRNA-214 expression is directly related to a healthy periodontal state and can be used as a reliable biomarker for periodontitis. miRNAs generally mediate vascular inflammation and apoptosis. Decrease in miRNA-214 expression was found to inhibit cell proliferation and induces apoptosis in vascular endothelial cells, specifically those induced by TNF-α [33]. This may explain the low levels of miRNA-214 in our periodontitis groups and highlight the effect of smoking on blood vessels, which caused more decreased levels in the smoker group than in the non-smoker group. Moreover, miRNA-214 showed significant negative correlation with probing depth in both periodontitis groups together and in nonsmoker periodontitis group alone. This enhances our rationalization that miRNA-214 under expression indicates disease.

In contradicting study, the gingival tissues of periodontally affected diabetic patients were examined and found that miRNA-214 levels were high in the periodontal tissue of diabetic participants. The authors explained this by stating that miRNA-214 plays a role in the regulation of necroptosis. Although this study opposes our findings, their results could be due to the inclusion of another factor, diabetes, that may affect miRNA-214 gene expression differently, while we studied smoking and searched for miRNA-214 expression in the GCF, not the gingival tissue [23].

In the herein study we found that smoking had a strong epigenetic impact on expression of our examined miRNAs (miRNA-214 &-223) where both were differently expressed with statistical significance in the smoker periodontitis group than healthy control, the first was under expressed while the second was overexpressed respectively. In a similar vein, Ongoz-Dede et al., in 2022 examined the saliva of smoker and non-smoker with gingivitis and periodontitis, as well as healthy participants. They observed an increase in salivary miRNAs [miRNA-146a, miRNA -146b, miRNA-203, and miR-155] as gingivitis progressed to periodontitis. [19].

In this study, we found that miRNA-214 is overexpressed in the healthy control group. In other words, its presence indicates healthy periodontium. In contrast, miRNA-223 was overexpressed in the nonsmoker and smoker periodontitis groups. This indicates that its presence indicates the disease state of the periodontium. Consistent with our outcomes,miRNA-223 is frequently identified in relation to periodontitis. MiRNA -223 and miRNA -200B have been found to have the highest expression levels in gingival tissues in periodontal disease [34]. Increased expression of miRNA -223 in inflamed periodontal tissues and its relation to osteoclastogenesis explains how it affects bone loss, which is a characteristic of periodontitis. MiRNA-223down regulation is directly related to decreased production of osteoclast [35]. On the other hand, and also in accordance with our results, four miRNAs, including miRNA-214, were significantly downregulated in the inflamed tissue [30, 34].

To the best of our knowledge, this study is the first to assess the diagnostic accuracy of miRNA-214 in GCF. Comparing both miRNA-214 & -223 for diagnostic accuracy, miRNA-214 yielded more accurate results, reaching 100% in the smoker periodontitis group and 97% in the nonsmoker periodontitis group. This opens the door for miRNA chair-side diagnostics to be able to detect the disease in its first steps and prevent unrepairable damage. In particular, it is noninvasive because it involves simply collecting the GCF.

MiRNA gene expression requires further investigation to clarify its role in healthy periodontal tissues and in diseased tissues. miRNAs mediate epigenetic changes in inflamed tissues and alveolar bone loss. A better understanding of these changes and their effects will lead to the development of alternative treatment modalities, besides helping to decrease edentulism due to periodontitis. This will pave the way for more precise and practical chairside diagnostics that can help identify the disease before damage occurs [30].

The limitations of this study include its small sample size. Hence, a larger sample size is required. Another limitation is the unifying of the severity of periodontal disease by adopting the same classification to properly compare the clinical severity of the disease.

## Conclusion

MiRNA-214 gene expression is abundant in healthy periodontal tissues. MiRNA-223 gene is overexpressed in patients with periodontitis, especially in smokers. Both miRNAs can be reliable diagnostic markers for periodontitis, with miRNA-214 being the most accurate.

## Footnotes

1 Note: this data is mandatory. Please provide.

2 MedCalc Software Ltd, Ostend, Belgium; https://www.medcalc.org; 2021.

3 R Core Team (2023). R: A language and environment for statistical computing. R Foundation for Statistical Computing, Vienna, Austria. URL https://www.R-project.org/.

## References

[1] Caton JG, Armitage G, Berglundh T et al (2018) A new classification scheme for periodontal and peri-implant diseases and conditions – Introduction and key changes from the 1999 classification. J Clin Periodontol 45(Suppl 20):S1–S8 29926489 10.1111/jcpe.12935

[2] Tonetti MS, Greenwell H, Kornman KS (2018) Staging and grading of periodontitis: framework and proposal of a new classification and case definition. J Periodontol 89(Suppl 1):S159–S172. 10.1002/JPER.18-0006. Erratum in: J Periodontol. 2018 Dec;89(12):1475 29926952 10.1002/JPER.18-0006

[3] Preshaw PM (2018) Host modulation therapy with anti-inflammatory agents. Periodontol 2000 76(1):131–149. 10.1111/prd.12148 29193331 10.1111/prd.12148

[4] Zhang J, Yu J, Dou J, Hu P, Guo Q (2021) The impact of smoking on subgingival plaque and the development of periodontitis: a literature review. Front Oral Health 2:751099. 10.3389/froh.2021.751099 35048061 10.3389/froh.2021.751099 PMC8757877

[5] Apatzidou DA (2022) The role of cigarette smoking in periodontal disease and treatment outcomes of dental implant therapy. Periodontol 2000 90(1):45–61. 10.1111/prd.12449 35950749 10.1111/prd.12449

[6] Bergström J, Eliasson S, Dock J (2000) Exposure to tobacco smoking and periodontal health. J Clin Periodontol 27(1):61–68. 10.1034/j.1600-051x.2000.027001061.x 10674963 10.1034/j.1600-051x.2000.027001061.x

[7] Palmer RM, Wilson RF, Hasan AS, Scott DA (2005) Mechanisms of action of environmental factors–tobacco smoking. J Clin Periodontol 32(Suppl 6):180–195. 10.1111/j.1600-051X.2005.00786.x 16128837 10.1111/j.1600-051X.2005.00786.x

[8] Théry C (2011) Exosomes: secreted vesicles and intercellular communications. F1000 Biol Rep 3:15. 10.3410/B3-15 21876726 10.3410/B3-15 PMC3155154

[9] Yoneda T, Tomofuji T, Ekuni D, Azuma T, Maruyama T, Fujimori K, Sugiura Y, Morita M (2019) Serum microRNAs and chronic periodontitis: a case-control study. Arch Oral Biol 101:57–63. 10.1016/j.archoralbio.2019.03.009 30889506 10.1016/j.archoralbio.2019.03.009

[10] Santonocito S, Polizzi A, Palazzo G, Isola G (2021) The emerging role of microRNA in periodontitis: pathophysiology, clinical potential and future molecular perspectives. Int J Mol Sci 22:5456. 10.3390/ijms22115456 34064286 10.3390/ijms22115456 PMC8196859

[11] Fulci V, Scappucci G, Sebastiani GD, Giannitti C, Franceschini D, Meloni F, Colombo T, Citarella F, Barnaba V, Minisola G, Galeazzi M, Macino G (2010) miR-223 is overexpressed in T-lymphocytes of patients affected by rheumatoid arthritis. Hum Immunol 71(2):206–211. 10.1016/j.humimm.2009.11.008 19931339 10.1016/j.humimm.2009.11.008

[12] Yu S, Zhao N, He M, Zhang K, Bi X (2020) MiRNA-214 promotes the pyroptosis and inhibits the proliferation of cervical cancer cells via regulating the expression of NLRP3. Cell Mol Biol (Noisy-le-grand) 66(6):59–64 33040786

[13] Yang S, Fei X, Lu Y, Xu B, Ma Y, Wan H (2019) miRNA-214 suppresses oxidative stress in diabetic nephropathy via the ROS/Akt/mTOR signaling pathway and uncoupling protein 2. Exp Ther Med 17(5):3530–3538. 10.3892/etm.2019.7359 30988734 10.3892/etm.2019.7359 PMC6447795

[14] Kuwabara Y, Ono K, Horie T, Nishi H, Nagao K, Kinoshita M, Watanabe S, Baba O, Kojima Y, Shizuta S, Imai M, Tamura T, Kita T, Kimura T (2011) Increased microRNA-1 and microRNA-133a levels in serum of patients with cardiovascular disease indicate myocardial damage. Circ Cardiovasc Genet 4(4):446–454. 10.1161/CIRCGENETICS.110.958975 21642241 10.1161/CIRCGENETICS.110.958975

[15] Yang Z, Chen H, Si H et al (2014) Serum miR-23a, a potential biomarker for diagnosis of pre-diabetes and type 2 diabetes. Acta Diabetol 51:823–831. 10.1007/s00592-014-0617-8 24981880 10.1007/s00592-014-0617-8

[16] Tomofuji T, Yoneda T, Machida T, Ekuni D, Azuma T, Kataoka K, Maruyama T, Morita M (2016) MicroRNAs as serum biomarkers for periodontitis. J Clin Periodontol 43(5):418–425. 10.1111/jcpe.12536 26910654 10.1111/jcpe.12536

[17] Amaral SA, Pereira TSF, Brito JAR et al (2019) Comparison of miRNA expression profiles in individuals with chronic or aggressive periodontitis. Oral Dis 25:561–568 30350903 10.1111/odi.12994

[18] Lee NH, Lee E, Kim YS, Kim WK, Lee YK, Kim SH (2020) Differential expression of microRNAs in the saliva of patients with aggressive periodontitis: a pilot study of potential biomarkers for aggressive periodontitis. J Periodontal Implant Sci 50(5):281–290. 10.5051/jpis.2000120006 33124206 10.5051/jpis.2000120006 PMC7606899

[19] Öngöz Dede F, Gökmenoğlu C, Türkmen E, Bozkurt Doğan Ş, Ayhan BS, Yildirim K (2023) Six miRNA expressions in the saliva of smokers and non-smokers with periodontal disease. J Periodontal Res 58(1):195–203. 10.1111/jre.13081 36495003 10.1111/jre.13081

[20] Bostanci N, Bao K (2017) Contribution of proteomics to our understanding of periodontal inflammation. Proteomics 17(3–4). 10.1002/pmic.201500518 10.1002/pmic.201500518 27995754

[21] Isola G, Santonocito S, Distefano A, Polizzi A, Vaccaro M, Raciti G, Alibrandi A, Li VG (2023) Impact of periodontitis on gingival crevicular fluid miRNAs profiles associated with cardiovascular disease risk. J Periodontal Res 58(1):165–174. 10.1111/jre.13078 36482859 10.1111/jre.13078

[22] Elazazy O, Amr K, Abd El Fattah A, Abouzaid M (2021) Evaluation of serum and gingival crevicular fluid microRNA-223, microRNA-203 and microRNA-200b expression in chronic periodontitis patients with and without diabetes type 2. Arch Oral Biol 121:104949. 10.1016/j.archoralbio.2020.104949 33157494 10.1016/j.archoralbio.2020.104949

[23] Ou L, Sun T, Cheng Y, Huang L, Zhan X, Zhang P, Yang J, Zhang Y, Zhou Z (2019) MicroRNA-214 contributes to regulation of necroptosis via targeting ATF4 in diabetes-associated periodontitis. J Cell Biochem 120(9):14791–14803. 10.1002/jcb.28740 31090954 10.1002/jcb.28740

[24] Silness J, Loe H (1964) Periodontal disease in pregnancy. Ii. Correlation between oral hygiene and periodontal condtion. Acta Odontol Scand 22:121–35. 10.3109/00016356408993968 14158464 10.3109/00016356408993968

[25] Glavind L, Löe H (1967) Errors in the clinical assessment of periodontal destruction. J Periodontal Res 2(3):180–184. 10.1111/j.1600-0765.1967.tb01887.x 4237475 10.1111/j.1600-0765.1967.tb01887.x

[26] Löe H (1967) The gingival index, the plaque index and the retention index systems. J Periodontol 38(6):Suppl:610-Suppl:616. 10.1902/jop.1967.38.6.610 5237684 10.1902/jop.1967.38.6.610

[27] Caton J (1989) Periodontal diagnosis and diagnostic aids: consensus report in Proceedings of the world workshop in clinical periodontics. Am Acad Periodontol. 10.1111/j.1834-7819.2009.01140.x

[28] Livak KJ, Schmittgen TD (2001) Analysis of relative gene expression data using real-time quantitative PCR and the 2(-Delta Delta C(T)) Method Methods 25:402–408. 10.1006/meth.2001.1262 10.1006/meth.2001.1262 11846609

[29] Papapanou PN, Sanz M, Buduneli N, Dietrich T, Feres M, Fine DH, Flemmig TF, Garcia R, Giannobile WV, Graziani F, Greenwell H, Herrera D, Kao RT, Kebschull M, Kinane DF, Kirkwood KL, Kocher T, Kornman KS, Kumar PS, Loos BG, Machtei E, Meng H, Mombelli A, Needleman I, Offenbacher S, Seymour GJ, Teles R, Tonetti MS (2018) Periodontitis: consensus report of workgroup 2 of the 2017 World Workshop on the classification of periodontal and Peri-implant diseases and conditions. J Periodontol 89(Suppl 1):S173–S182. 10.1002/JPER.17-0721 29926951 10.1002/JPER.17-0721

[30] Irwandi RA, Vacharaksa A (2016) The role of microRNA in periodontal tissue: a review of the literature. Arch Oral Biol 72:66–74. 10.1016/j.archoralbio.2016.08.014 27552373 10.1016/j.archoralbio.2016.08.014

[31] Cheung HS, Carballosa C, Greenberg J (2019) Role of MicroRNA in smoking–induced periodontitis. In Non-Coding RNAs.IntechOpen. 10.5772/intechopen.86756

[32] Zhang Z, Wang M, Zheng Y, Dai Y, Chou J, Bian X, Wang P, Li C, Shen J (2022) MicroRNA-223 negatively regulates the osteogenic differentiation of periodontal ligament derived cells by directly targeting growth factor receptors. J Transl Med 20(1):465. 10.1186/s12967-022-03676-1 36221121 10.1186/s12967-022-03676-1 PMC9552407

[33] Wang M, Liu M, Ni T, Liu Q (2018) miR-214 mediates vascular inflammation and apoptosis via PTEN expression. Mol Med Rep 18(2):2229–2236. 10.3892/mmr.2018.9185 29916551 10.3892/mmr.2018.9185

[34] Ogata Y, Matsui S, Kato A, Zhou L, Nakayama Y, Takai H (2014) MicroRNA expression in inflamed and noninflamed gingival tissues from Japanese patients. J Oral Sci 56(4):253–260 25500922 10.2334/josnusd.56.253

[35] Sugatani T, Hruska KA (2009) Impaired micro-RNA pathways diminish osteoclast differentiation and function. J Biol Chem 284(7):4667–4678. 10.1074/jbc.M805777200 19059913 10.1074/jbc.M805777200 PMC2640963

